# Association Between Systemic Immune Inflammation Index and Diabetes Mellitus in the NHANES 2003-2018 Population

**DOI:** 10.1210/jendso/bvae124

**Published:** 2024-06-27

**Authors:** Yufeng Yan, Hongjing Lu, Yaguo Zheng, Song Lin

**Affiliations:** Department of Cardiology, Nanjing First Hospital, Nanjing Medical University, Nanjing 210008, Jiangsu, China; Department of Cardiology, Nanjing First Hospital, Nanjing Medical University, Nanjing 210008, Jiangsu, China; Department of Cardiology, Nanjing First Hospital, Nanjing Medical University, Nanjing 210008, Jiangsu, China; Department of Cardiology, Nanjing First Hospital, Nanjing Medical University, Nanjing 210008, Jiangsu, China

**Keywords:** diabetes mellitus, Systemic Immune-Inflammation Index, NHANES mortality

## Abstract

**Objects:**

This study aimed to explore the association between the Systemic Immune-Inflammation Index (SII) and diabetes mellitus (DM) and to assess its influence on the prognosis of the DM and no-DM groups.

**Methods:**

The study used data from the National Health and Nutrition Examination Survey; 9643 participants were included. Logistic regression analysis was employed to evaluate connections between SII and DM. We used the Cox proportional hazards model, restricted cubic spline, and Kaplan–Meier curve to analyze the relationship between SII and mortality.

**Results:**

The logistic regression analysis indicated that a significant increase in the likelihood of developing DM with higher SII levels (odds ratio, 1.31; 95% CI, 1.09-1.57, *P* = .003). The Cox model showed that there is a positive association between increased SII and higher all-cause mortality. The hazard ratios for SII were 1.53 (1.31, 1.78), 1.61 (1.31, 1.98), and 1.41 (1.12, 1.78) in the total, DM and non-DM groups, respectively. We observed a linear correlation between SII and all-cause mortality in DM participants, whereas non-DM participants and the total population showed a nonlinear correlation.

**Conclusion:**

Elevated SII levels are linked to an augmented risk of DM. Those with DM and higher SII levels demonstrated an elevated risk of mortality.

## Background

Diabetes mellitus (DM) is a common endocrine system disorder characterized by inadequate insulin secretion or persistently high blood glucose levels. DM affects approximately 500 million individuals and is projected to increase to 738 million people by 2045 [1]. This condition bears significant implications for human health, leading to diminished life expectancy [2] Notably, cardiovascular disease (CVD) emerges as the primary cause of morbidity and mortality in those with type 2 DM, substantially heightening the risk of cardiovascular morbidity [3]. The escalating prevalence of type 2 diabetes has consequently contributed to increased mortality rates among young and middle-aged adults. The etiology of DM is multifaceted, encompassing factors such as immune response, genetic predisposition, and environmental influences [4].

The Systemic Immune-Inflammatory Index (SII) represents a novel biomarker assessing immune-inflammatory responses in humans, initially proposed in 2014 to predict prognosis in individuals undergoing radical resection for liver cancer [5]. The SII, formulated by Hu and colleagues in 2014, has undergone widespread scrutiny and thorough exploration in subsequent investigations. The index is derived by multiplying the platelet count by the neutrophil/lymphocyte ratio. Extensive research has been conducted on the SII, which has been linked to the emergence of multiple illnesses, such as cancer [6], periodontitis [7], coronary heart disease [8], low muscle mass [9], and chronic obstructive pulmonary disease [10]. The SII is not only associated with disease onset but also closely linked to the prognosis of conditions such as heart failure [11], hypertension [12], and pancreatic cancer [13]. Many research studies have consistently demonstrated a notable correlation between inflammation and the onset of DM [14, 15]. Regulatory T-lymphocyte cells can be used to treat autoimmune diabetes in a basic study [16]. Chronic tissue inflammation plays a vital role in metabolic diseases [17-19]. Some studies found that adipose tissue macrophages contribute to the insulin-resistant state [20, 21]. It is widely believed that macrophages are the primary effector cells responsible for reduced insulin signaling [22]. Immune cell types contribute to the development of insulin resistance and type 2 diabetes in individuals by participating in adipose tissue inflammation [23]. Adipose tissue macrophages produce factors that disrupt insulin signaling in target cells. These factors act in a paracrine or systemic manner [24].

However, research examining the association between DM and SII remains limited, with insufficient evidence to establish a definitive link between SII and all-cause mortality or specific causes of death in patients with DM. This study aims to address this gap by using data from The National Health and Nutrition Examination Survey (NHANES) to analyze the relationship between SII and DM and evaluate SII's impact on mortality rates within cohorts with and without diabetes.

## Methods

### Study Design and Population

**Figure 1.**
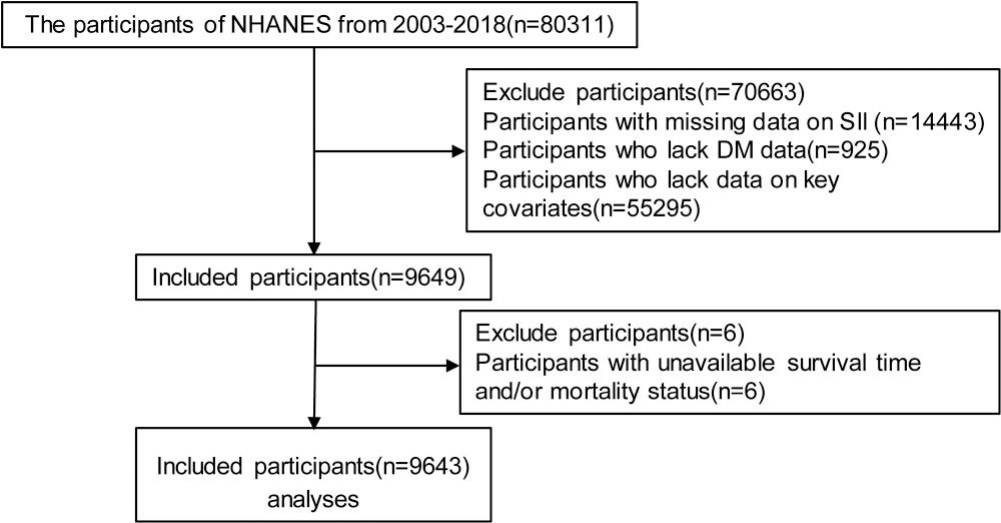
The flow chart of participant selection.

The NHANES is a major epidemiological survey conducted by the National Center for Health Surveys of the US Department of Health and Human Services. It aims to evaluate the health and nutritional status of the US population, providing vital data to policymakers, researchers, and the public [25]. The research protocol of NHANES was approved by the Ethical Review Committee of the National Center for Health Surveys. All participants in this study provided written informed consent. Data from NHANES 2003-2018 were included in this study, encompassing a total of 80 311 individuals. We excluded 14 443 participants for a missing SII, 925 for missing DM, and 55 295 for missing other key variables including age, male, poverty income ratio, body mass index (BMI), race, education level, smoking, alcohol drinks, low-density lipoprotein cholesterol (LDL-C), high-density lipoprotein cholesterol (HDL-C), total cholesterol (TC) and total triglyceride (TG), systolic blood pressure (SBP), and diastolic blood pressure (DBP). We then further excluded 6 participants for whom we lacked unavailable survival time or mortality data, resulting in a final study population of 9643 individuals for analysis. Figure 1 provides a flow chart of participant selection.

#### Covariates

Standardized questionnaires were used to gather information on age, gender, race/ethnicity, education level, smoking habits, alcohol consumption, and poverty income ratio. Race was categorized as non-Hispanic white, non-Hispanic Black, Mexican American, Other Hispanic, or others. Educational background was classified as grades 9-11, some college, college graduate, or high school graduate. The leukocyte platelet count, neutrophil count, lymphocyte count, LDL-C, HDL-C, TC, and TG were ascertained via standard biochemistry profiling. Examination components provided the SBP, DBP, and BMI. DM was characterized by self-reported physician diagnosis, HbA1c ≥ 6.5%, fasting plasma glucose ≥ 7 mmol/L, 2-hour oral glucose tolerance test plasma glucose ≥11.1 mmol/L, or diabetes medication use. Participants also reported their medical history including previous occurrences of DM, hypertension, coronary heart disease, congestive heart failure, stroke, heart attack, and angina. The SII index was calculated as the product of platelet count, neutrophil count, and lymphocyte count divided by the preoperative lymphocyte count: SII = (peripheral platelet count × neutrophil count)/lymphocyte count.

#### Outcomes and follow up

The outcomes and follow up of the study were required by the NHANES public use-linked mortality file with follow-up conducted until December 31, 2019. The primary endpoint was defined as all-cause mortality. Cardiovascular mortality and cardiovascular and cerebrovascular mortality were defined as the secondary endpoints. Cardiovascular mortality was defined using International Classification of Diseases-10 codes 100-109, 111, 113, and 120-I51. Cardiovascular and cerebrovascular mortality was defined using International Classification of Diseases-10 codes 100-109, 111, 113, 120-151, or 160-169.

### Statistical Analysis

In accordance with previous research [7, 8], the baseline variables were equally divided into 4 groups based on the level of SII. These groups were categorized as follows: SII (Q1, ≤322.36), low-moderate SII (Q2, 322.36-447.70), intermediate SII (Q3, 447.70-633.11), and high SII (Q4, >633.11). Differences among the groups were analyzed using 1-way analysis of variance and chi-square tests. Continuous variables were summarized using mean ± SD or median with interquartile range, whereas categorical variables were presented as percentages or numbers (percentage). To assess the relationship between DM and SII levels, multivariate logistic regression models were employed, with odds ratios and CIs presented as results. Model 1 represented the unadjusted data. In Model 2, the data were adjusted for age, sex, and race. Model 3 adjusted variables as follows: age, sex, poverty income ratio, BMI, race, education level, smoking status, alcohol consumption, TG, TC, HDL, LDL, SBP, and DBP. Restricted cubic plots were employed to explore whether there was a nonlinear relationship between SII and DM adjusted in model 3. Additionally, the Kaplan–Meier curve was performed to analysis the event-free survival rates of the groups. We used multivariate Cox proportional regression models to investigate the relationships between levels of SII and the risks of mortality. The models were adjusted for age, sex, BMI, race, poverty income ratio, education level, smoking status, alcohol consumption, TG, TC, HDL, LDL, SBP, DBP, and CVD. The hazard ratio (HR) and 95% CIs were computed using multivariable Cox regression analyses. After adjusting for the previously mentioned variables, we examined the potential nonlinear association between SII and mortality risk using restricted cubic splines. All analysis methods were used in the whole group, the group with diabetes, and the group without diabetes. All statistical analyses were conducted using R, version 4.2.0. *P* < .05 was considered significant.

## Results

### Baseline Characteristics

The study cohort included 1533 participant with DM (15.9%) and 8110 participants without DM (84.1%). When comparing the participants in the lower SII group to those in the SII Q4 (highest quartile) group, several differences were observed. The participants in the SII Q4 group had a lower proportion of males, a higher proportion of non-Hispanic White individuals, a higher proportion of current smokers, a higher proportion of individuals with lower education levels, a higher proportion of alcohol consumers, a higher proportion with DM, and higher levels of BMI, TG, TC, HDL, and SBP (*P* < .05). Table 1 shows the basic characteristics of participants. Table 2 shows baseline characteristics between the DM and no-DM groups. The DM group has a higher proportion of males, current smokers, CVD, and a higher level of age, BMI, SII, TG, TC, HDL, SBP, and DBP (*P* < .05).

**Table 1.** Patient demographics and baseline characteristics

Variable	SII				*P* value
	Quartile 1	Quartile 2	Quartile 3	Quartile 4	
	N = 2410	N = 2411	N = 2411	N = 2411	
Age, y	46.4 (17.1)	46.2 (16.7)	47.1 (16.9)	49.2 (17.5)	**<.001**
Male, %	1439 (59.7%)	1382 (57.3%)	1255 (52.1%)	1149 (47.7%)	**<**.**001**
Poverty income ratio	2.7 (1.6)	2.8 (1.7)	2.82 (1.7)	2.7 (1.6)	**<**.**001**
Race/ethnicity, %					**<**.**001**
Non-Hispanic White	901 (37.4%)	1167 (48.4%)	1234 (51.2%)	1350 (56.0%)	
Non-Hispanic Black	685 (28.4%)	390 (16.2%)	386 (16.0%)	343 (14.2%)	
Other race	285 (11.8%)	225 (9.3%)	205 (8.5%)	185 (7.7%)	
Mexican American	348 (14.4%)	406 (16.8%)	362 (15.0%)	342 (14.2%)	
Other Hispanic	191 (7.9%)	223 (9.2%)	224 (9.3%)	191 (7.9%)	
Education level, %					.**004**
High school graduate	557 (23.1%)	489 (20.3%)	542 (22.5%)	585 (24.3%)	
<9th grade	154 (6.4%)	169 (7.0%)	155 (6.4%)	162 (6.7%)	
Some college	759 (31.5%)	732 (30.4%)	784 (32.5%)	771 (32.0%)	
College graduate	658 (27.3%)	724 (30.0%)	633 (26.3%)	584 (24.2%)	
Grade 9-11	282 (11.7%)	297 (12.3%)	297 (12.3%)	309 (12.8%)	
Smoke					**<**.**001**
Never	1305 (54.1%)	1255 (52.1%)	1226 (50.9%)	1074 (44.5%)	
Now	505 (21.0%)	513 (21.3%)	562 (23.3%)	687 (28.5%)	
Former	600 (24.9%)	643 (26.7%)	623 (25.8%)	650 (27.0%)	
Alcohol drinks/day	2.7 (2.4)	2.9 (2.7)	2.7 (2.5)	2.7 (2.9)	.053
BMI, kg/m^2^	27.8 (6.0)	28.5 (6.2)	29.2 (6.7)	29.8 (7.9)	**<**.**001**
DM, %	348 (14.4%)	349 (14.5%)	382 (15.8%)	454 (18.8%)	**<**.**001**
CVD, %	198 (8.2%)	185 (7.7%)	195 (8.1%)	262 (10.9%)	**<**.**001**
SII, 1000 cells/µL	241.3 (59.7)	383.6 (35.5)	532.4 (52.9)	922.2 (358.9)	**<**.**001**
TG, mg/dL	111.9 (65.8)	118.3 (67.4)	120.7 (67.4)	119.8 (64.9)	**<**.**001**
TC, mg/dL	190.7 (39.4)	193.7 (40.7)	193.1 (39.7)	192.1 (39.6)	.**048**
HDL, mg/dL	55.7 (16.8)	54.7 (16.3)	54.4 (16.4)	54.4 (15.3)	.**01**
LDL, mg/dL	112.8 (34.3)	115.3 (35.5)	114.6 (34.7	113.8 (35.3)	.063
SBP, mm Hg	121.3 (17.7)	121.7 (17.0)	121.4 (17.1)	123.9 (17.8)	**<**.**001**
DBP, mm Hg	69.9 (11.3)	70.3 (11.4)	69.8 (11.7)	70.2 (11.9)	.391

Mean (SD) for continuous variables, % for categorical variables. Quartile 1: SII ≤ 322.36; Quartile 2: 322.36 < SII ≤ 447.70; Quartile 3: 447.70 <SII ≤ 633.11; Quartile 4: SII > 633.11. Bold values indicate a *P*-value less than .05.

Abbreviations: BMI, body mass index; CVD, cardiovascular disease; DBP, diastolic blood pressure; DM, diabetes mellitus; HDL-C, high-density lipoprotein cholesterol; LDL-C, low-density lipoprotein cholesterol; SBP, systolic blood pressure; SII, Systemic Immune-Inflammation Index; TC, total cholesterol; TG, total triglyceride.

**Table 2.** Baseline characteristics between the DM and no-DM groups

Variables	Total (n = 9643)	No-DM (n = 8110)	DM (n = 1533)	*P*
Age, y	47.2 (17.1)	45.0 (16.8)	58.9 (13.8)	**<**.**001**
Male, %	5225 (54.2%)	4315 (53.2%)	910 (59.4%)	**<**.**001**
Poverty income ratio	2.8 (1.6)	2.8 (1.7)	2.6 (1.6)	.**001**
Race/ethnicity, %				**<**.**001**
Non-Hispanic White	4652 (48.2%)	4015 (49.5%)	637 (41.6%)	
Non-Hispanic Black	1804 (18.7%)	1463 (18.0%)	341 (22.2%)	
Other race	900 (9.3%)	764 (9.4%)	136 (8.9%)	
Mexican American	1458 (15.1%)	1193 (14.7%)	265 (17.3%)	
Other Hispanic	829 (8.6%)	675 (8.3%)	154 (10.0%)	
Education level, %				**<**.**001**
High school graduate	2173 (22.5%)	1789 (22.1%)	384 (25.0%)	
<9th grade	640 (6.6%)	469 (5.8%)	171 (11.2%)	
Some college	3046 (31.6%)	2588 (31.9%)	458 (29.9%)	
College graduate	2599 (26.9%)	2283 (28.2%)	316 (20.6%)	
Grade 9-11	2173 (22.5%)	1789 (22.1%)	384 (25.0%)	
Smoke				**<**.**001**
Never	2516 (26.1%)	1990 (24.5%)	526 (34.3)	
Now	4860 (50.4%)	4174 (51.5%)	686 (44.7%)	
Former	2267 (23.5%)	1946 (23.9%)	321 (20.9%)	
Alcohol drinks/day	2.7 (2.6)	2.8 (2.6)	2.5 (2.4)	**<**.**001**
BMI, kg/m^2^	28.8 (6.8)	28.2 (6.5)	31.9 (7.4)	**<**.**001**
CVD, %	840 (8.7%)	515 (6.4%)	325 (21.2%)	**<**.**001**
SII, 1000 cells/µL	519.9 (314.1)	511.8 (297.9)	562.6 (385.8)	**<**.**001**
TG, mg/dL	117.4 (66.5)	112.9 (64.2)	141.4 (72.9)	**<**.**001**
TC, mg/dL	192.4 (39.9)	193.3 (39.2)	187.4 (43.2)	**<**.**001**
HDL, mg/dL	54.8 (16.2)	556 (16.1)	50.8 (16.2)	**<**.**001**
LDL, mg/dL	114.1 (34.9)	115.2 (34.4)	108.3 (37.4)	**<**.**001**
SBP, mm Hg	122.1 (17.4)	120.5 (16.7)	130.5 (18.9)	**<**.**001**
DBP, mm Hg	70.1 (11.6)	69.9 (11.4)	70.9 (12.4)	.**005**

Bold values indicate a *P*-value less than .05.

Abbreviations: BMI, body mass index; CVD, cardiovascular disease; DBP, diastolic blood pressure; DM, diabetes mellitus; HDL-C, high-density lipoprotein cholesterol; LDL-C, low-density lipoprotein cholesterol; SBP, systolic blood pressure; SII, Systemic Immune-Inflammation Index; TC, total cholesterol; TG, total triglyceride.

Mean (SD) for continuous variables, % for categorical variables.

### Relationship Between SII and DM

**Figure 2.**
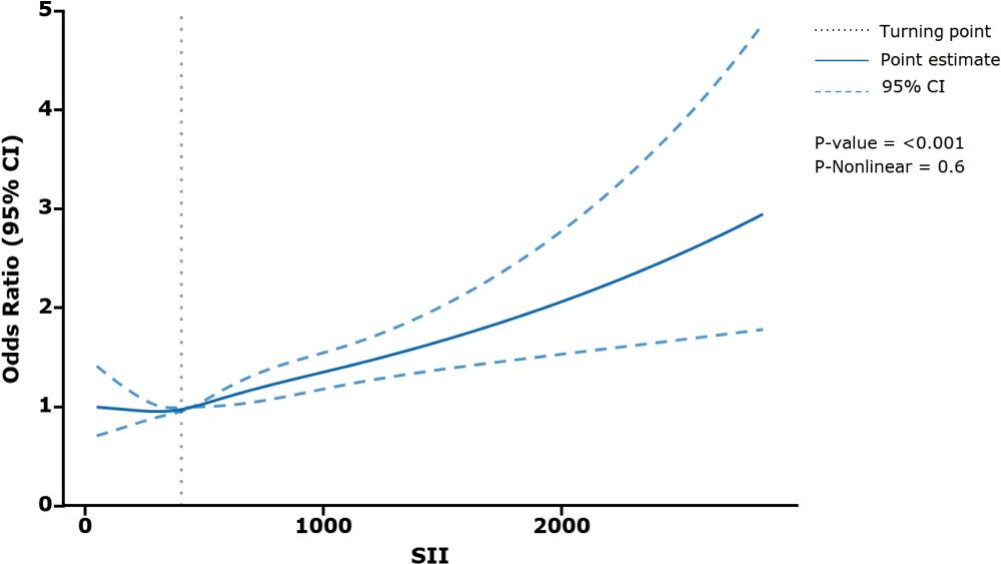
Restricted cube plots to evaluate the nonlinear relationship between SII and the risk of DM. SII, Systemic Immune-Inflammation Index.

Multivariable logistic regression models showed that there were positive associations between SII and DM after adjusted for the fully confounding variables (OR, 1.311; 95% CI, 1.094-1.570; *P* = .003). The results are presented in Table 3. Additionally, when analyzing SII as a categorical variable (divided into quartiles), all the multivariate logistic regression models indicated an escalating risk of DM in quartiles with elevated SII in comparison to the lowest quartile (*P* < .001). Figure 2 shows the relationship between SII and DM in restricted cubic plots adjusted in Model 3. There was a positive association between SII and diabetes that was shown in restricted cubic plots.

**Table 3.** The associations between SII and DM in the logistic regression models

SII	Model 1		Model 2		Model 3	
	OR (95% CI)	*P* value	OR (95% CI)	*P* value	OR (95% CI)	*P* value
SII Per 1000	1.571 (1.345-1.835)	**<.001**	1.056 (1.052-1.060)	**<**.**001**	1.311 (1.094-1.570)	.**003**
SII (Quartile)						
Q1	ref		ref		ref	
Q2	1.003 (0.854-1.178)	.972	0.694 (0.588-0.819)	**<**.**001**	1.057 (0.882-1.265)	.549
Q3	1.116 (0.953-1.306)	.174	0.774 (0.657-0.911)	.**002**	1.116 (0.934-1.333)	.226
Q4	1.375 (1.180-1.601)	**<**.**001**	0.863 (0.735-1.012)	.069	1.194 (1.003-1.422)	.**047**

Model 1 was unadjusted. Model 2 was adjusted for age, sex, and race. Model 3 was adjusted for age sex, poverty income ratio, body mass index, race, education level, smoking status, alcohol consumption, triglyceride, total cholesterol, high-density lipoprotein, low-density lipoprotein, systolic blood pressure, and diastolic blood pressure. Bold values indicate a *P*-value less than .05.

### Long-term Outcomes in Overall Participants

**Figure 3.**
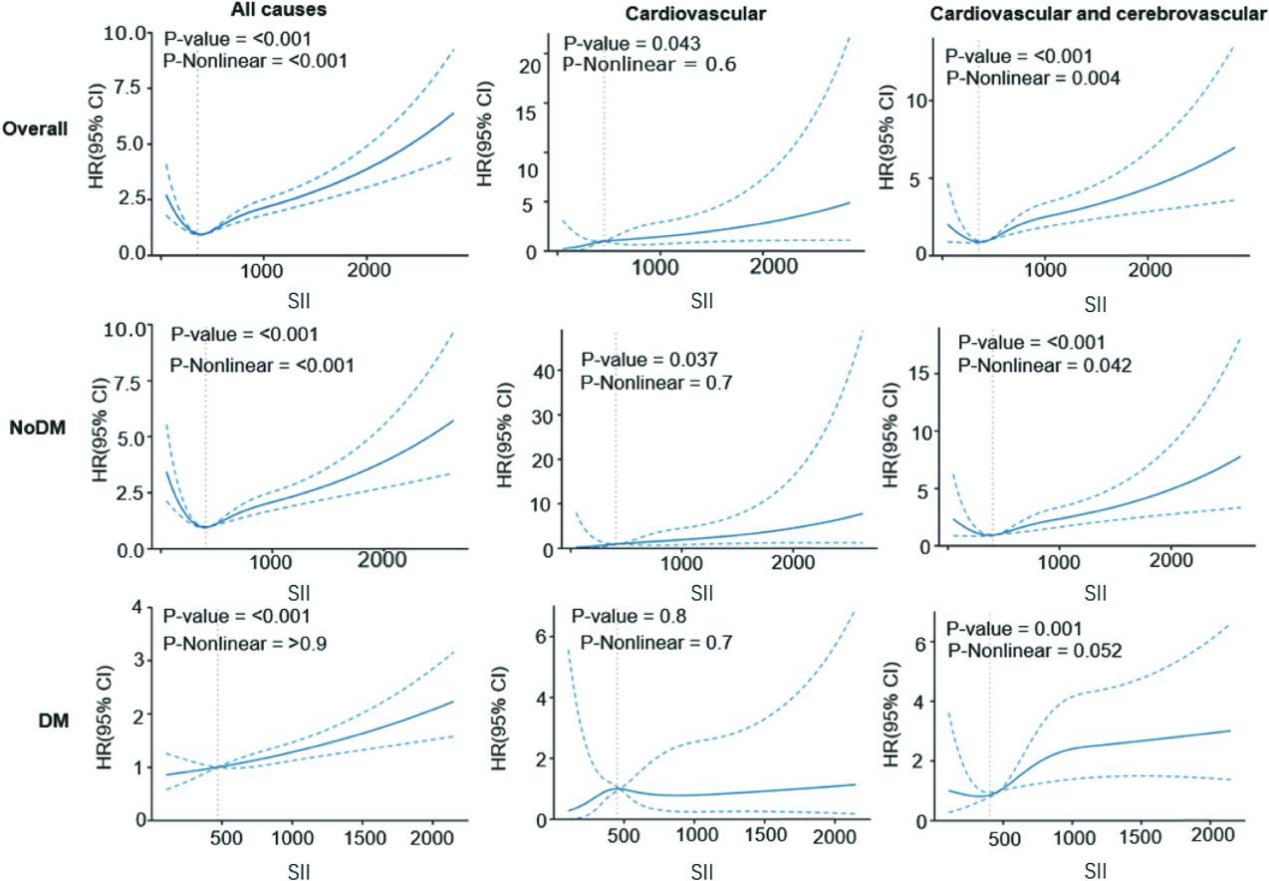
Restricted cube plots to evaluate the nonlinear relationship between SII and the risk of mortality. (A) All-cause mortality in overall patients. (B) Cardiovascular mortality in overall patients. (C) Cardiovascular and cerebrovascular mortality in overall patients. (D) All-cause mortality in no-DM patients. (E) Cardiovascular mortality in no-DM patients. (F) Cardiovascular and cerebrovascular mortality in no-DM patients. (G) All-cause mortality in DM patients. (H) Cardiovascular mortality in DM patients. (I) Cardiovascular and cerebrovascular mortality in DM patients. Q1: SII ≤ 322.36; Q2: 322.36 < SII ≤ 447.70; Q3: 447.70 <SII ≤ 633.11; Q4: SII > 633.11.

**Figure 4.**
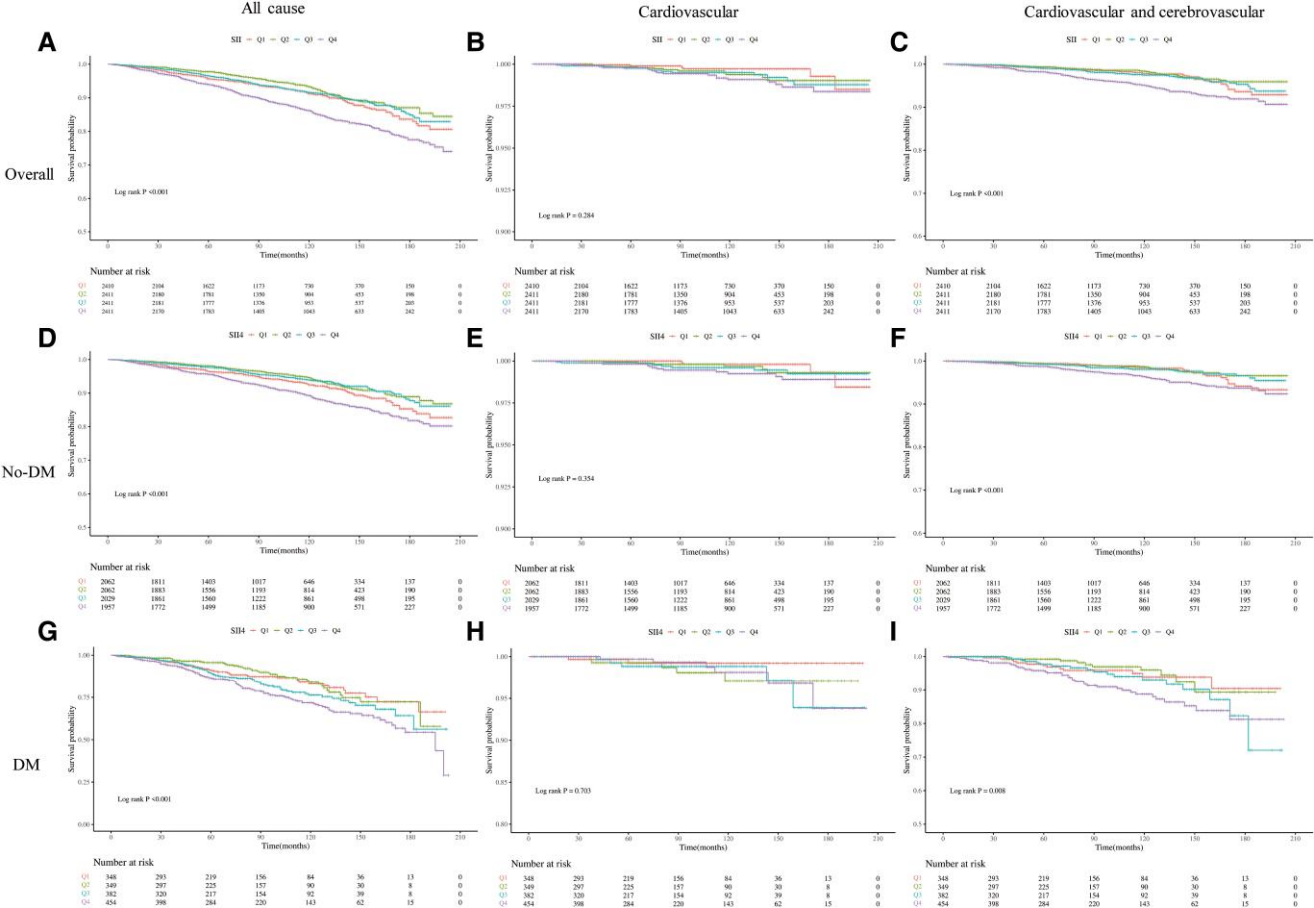
Kaplan–Meier curves illustrating the association of SII with all-cause mortality and specific cause. (A) All-cause mortality in overall patients. (B) Cardiovascular mortality in overall patients. (C) Cardiovascular and cerebrovascular mortality in overall patients. (D) All-cause mortality in no-DM patients. (E) Cardiovascular mortality in no-DM patients. (F) Cardiovascular and cerebrovascular mortality in no-DM patients. (G) All-cause mortality in DM patients. (H) Cardiovascular mortality in DM patients. (I)cardiovascular and cerebrovascular mortality in DM patients. Q1: SII ≤ 322.36; Q2: 322.36 < SII ≤ 447.70; Q3: 447.70 <SII ≤ 633.11; Q4: SII > 633.11.

During the average 105 months of follow up, 840 participants (8.71%) experienced all-cause mortality, with 262 having DM. After employing fully adjusted Cox proportional regression models (Table 4), we discovered an association between a higher SII and an increased risk of mortality from all causes (*P* < .001) as well as mortality from cardiovascular causes (*P* = .039) and mortality from cardiovascular and cerebrovascular causes (*P* < .001). Furthermore, using restrictive cubic spline regression (Fig. 3), we identified a nonlinear connection between different levels of SII and the risk of all-cause mortality when adjusting for confounding variables (*P* < .001). This nonlinear correlation was also evident for mortality pertaining to cardiovascular and cerebrovascular reasons (*P* = .004). However, there was no evidence of a nonlinear association with cardiovascular mortality (*P* = .6). We assessed the HR for all-cause mortality in people belonging to the fourth quartile of SII compared to those in the second group, while accounting for confounding variables. The multivariable adjusted hazard ratio was 1.09 (95% CI, 1.08-1.10; *P* < .001). A meaningful correlation was also observed between the uppermost quartile of SII and the probability of death from cardiovascular and cerebrovascular causes (HR, 1.59; 95% CI, 1.11-2.29; *P* = .012). Compared with the second group, there was no significant difference between other groups and cardiovascular mortality in the overall group. On reviewing the Kaplan–Meier curves in the entire sample (Fig. 4), it was apparent that participants in the lowest quartile of SII possessed a reduced risk of all-cause mortality (*P*_log-rank_ < .001) and mortality resulting from cardiovascular and cerebrovascular causes (*P*_log-rank_ < .001). However, no significant differences were detected for cardiovascular mortality (*P*_log-rank_ = .06).

**Table 4.** Association of SII with all-cause and cause specific mortality in Cox proportional regression models

SII	All cause		Cardiovascular		Cardiovascular and cerebrovascular	
	HR (95% CI)	*P* value	HR (95% CI)	*P* value	HR (95% CI)	*P* value
Overall patients						
SII Per 1000	1.53 (1.31-1.78)	**<.001**	1.88 (1.03-3.44)	.**039**	1.86 (1.46-2.36)	**<**.**001**
SII (quartile)						
Q1	ref					
Q2	0.86 (0.69-1.07)	.18	2.34 (0.84-6.53)	.103	0.95 (0.62-1.45)	.804
Q3	0.85 (0.69-1.05)	.135	2.25 (0.83-6.09)	.109	1.01 (0.68-1.50)	.974
Q4	1.12 (0.93-1.35)	.247	2.01 (0.78-5.22)	.15	1.51 (1.06-2.15)	.**022**
No-DM						
SII per 1000	1.41 (1.12-1.78)	.**003**	3.12 (1.38-7.08)	.**006**	1.79 (1.04-3.07)	.**034**
SII (Quartile)						
Q1	ref					
Q2	0.91 (0.70-1.17)	.468	1.96 (0.52-7.43)	.324	1.07 (0.65-1.77)	.783
Q3	0.76 (0.59-0.97)	.**029**	2.32 (0.65-8.28)	.194	0.95 (0.59-1.54)	.83
Q4	1.04 (0.83-1.30)	.738	2.41 (0.73-7.98)	.151	1.43 (0.93-2.19)	.1
DM						
SII per 1000	1.61 (1.31-1.98)	**<**.**001**	1.18 (0.31-4.48)	.803	1.78 (1.21-2.61)	.003
SII (quartile)						
Q1	ref					
Q2	0.69 (0.45-1.06)	.091	1.49 (0.26-8.60)	.654	0.59 (0.26-1.38)	.225
Q3	1.06 (0.72-1.57)	.769	2.26 (0.37-13.68)	.376	1.13 (0.53-2.37)	.756
Q4	0.84 (0.55-1.27)	.403	0.99 (0.11-9.02)	.996	1.21 (0.55-2.67)	.64

Q1: SII ≤ 322.36; Q2: 322.36 < SII ≤ 447.70; Q3: 447.70 <SII ≤ 633.11; Q4: SII > 633.11. Bold values indicate a *P*-value less than .05.

### Long-term Outcomes in Participants With no DM

In the no-DM group, Kaplan–Meier curves showed a significant difference in all-cause mortality risk (*P*_log-rank_ < .001) and cardiovascular and cerebrovascular mortality (*P*_log-rank_ < .001) among the 4 groups. Results indicate that there were no major differences in the incidence of cardiovascular mortality among participants without diabetes across the 4 cohorts (*P*_log-rank_ = .35). In fully adjusted Cox proportional regression models, increased levels of SII displayed an association with augmented mortality risk from any cause (*P* = .003), amplified risk of mortality from cardiovascular issues (*P* = .006), and increased risk of mortality resulting from cardiovascular and cerebrovascular problems (*P* = .034) solely in the no-DM group.

Compared with reference group, there was no difference for between the other quartile of SII regardless of the risk of outcome. Restricted cubic spline regression analysis demonstrated a nonlinear relationship between SII levels and the risk of mortality from any cause (*P* < .001), as well as cardiovascular and cerebrovascular mortality (*P* = .042) in the no-DM group.

### Long-term Outcomes in Participants With DM

In the DM group, Kaplan–Meier curves demonstrated a significant difference in the risk of all-cause mortality (*P*_log-rank_ < .001) and cardiovascular and cerebrovascular mortality (*P*_log-rank_ = .008) among the 4 types of individuals (SII quartile 1, quartile 2, quartile 3, quartile 4). However, no significant correlation was found between the 4 SII quartiles and the risk of cardiovascular-related death (*P*_log-rank_ = .7). In fully adjusted models using Cox proportional regression, increased levels of SII were linked to a greater risk of mortality from all causes (*P* < .001), as well as mortality related to cerebrovascular and cardiovascular conditions (*P* = .003) in the DM group. There was, however, no noticeable difference found in cardiovascular mortality (*P* = .803). On analyzing variables, and comparing to reference, individuals in the third quartile had an adjusted hazard ratio for all-cause mortality of 1.65 (95% CI, 1.10-2.46; *P* = .015), whereas individuals in the fourth quartile had an adjusted HR of 1.68 (95% CI, 1.16-2.42; *P* = .006), as shown by multivariable analysis accounting for confounding covariates. The use of restricted cubic spline regression analysis revealed a nonlinear association between SII levels and the risk of all-cause mortality (*P* = .9), cardiovascular mortality (*P* = .7), as well as cardiovascular and cerebrovascular mortality (*P* = .052) after adjustment for confounding variables (*P* < .001).

### The Difference of Long-term Outcomes Between DM Patients and No-DM Patients

DM patients has higher level of the SII than no-DM patients. For CVD-related mortality, there is no significant difference between level of the SII with cardiovascular mortality in those with DM (HR, 1.18; 95% CI, 0.31-4.48; *P* = .803). However, there is a significant difference cardiovascular mortality between no-DM patients with DM patients. For the cardiovascular and cerebrovascular related mortality, the no-DM patients has a higher HR than DM patients (1.79 vs 1.78).

## Discussion

Our study suggests a linear relationship between SII and DM, perhaps providing a guide for early identification of DM participants. We further explored the prognostic impact of SII in both DM participants and no-DM participants.

The pathophysiology of diabetes involves chronic inflammatory responses intricately linked to the emergence of complications such as diabetic retinopathy [26], microangiopathy [27], and diabetic nephropathy [28]. For instance, in diabetic retinopathy, elevated inflammatory cytokines and monocyte infiltration contribute to disease progression [29]. Similarly, macrophage infiltration into the kidneys promotes inflammation and fibrosis in diabetic nephropathy, leading to kidney damage and dysfunction [30]. Neutrophils and platelets are involved in the inflammatory and wound healing response in participants with diabetes, and studies have shown that participants with diabetes have platelet dysfunction and thus reduced wound-healing capacity [31].

Improvements in hematological inflammatory biomarkers have been associated with improved glycemia in individuals with diabetes. A clinical study has shown that leukocyte and neutrophil counts, main platelet volume, and platelet distribution width values were significantly higher in individuals with type 2 DM who were receiving medication compared to individuals in the healthy control group [32]. Combinations of inflammatory ratios, including the neutrophil-to-lymphocyte ratio (NLR) and platelet-to-lymphocyte ratio (PLR), have been found to have a close association with the prognosis of diabetes and are predictive in individuals with diabetes. Studies have shown that NLR significantly increases in those with prediabetes and diabetes, whereas PLR increases in the early stages of diabetes and decreases in the late stages [33]. Inflammatory ratios such as NLR and PLR show promise as prognostic indicators in diabetes, with NLR significantly increasing in those with prediabetes and diabetes and PLR exhibiting stage-specific variations. These markers also correlate with adverse clinical outcomes in diabetic nephropathy and coronary artery disease patients undergoing PCI, highlighting their predictive value in diabetes management [33-35]. SII is a novel inflammatory biomarker that combines neutrophil, platelet, and lymphocyte counts and has been shown to correlate with the prognosis and development of several diseases [7, 36]. We found that an association between SII index and DM after adjusting for other variables. A recent study showed an association between the SII and diabetes [37]. In addition to exploring the relationship between SII and DM, our study explored the impact of the SII on the death of DM patients. Some study found SII can better predict the prognosis of diseases compared to other inflammatory indicators such as NLR and PLR [38, 39]. Further research is needed to compare difference between SII with other DM monitoring markers in the diagnose or prognose.

Inflammatory markers have a crucial role in patient prognosis, as evidenced by several studies indicating that elevated SII levels are associated with a higher risk of all-cause mortality [7, 40, 41]. Our study reached similar conclusions. We examined the association between SII and mortality in both populations with and without diabetes. Within the group with diabetes, the elevated SII subgroups had a greater mortality risk in contrast to their nondiabetic counterparts. Additionally, there was no higher risk of cardiovascular death observed in higher SII subgroups within both populations with and without diabetes. This finding contrasts with a recent meta-analysis demonstrating the prognostic value of SII for cardiovascular death in coronary artery disease participants [42]. A prospective study involving 1202 participants revealed that SII was a robust predictor of adverse cardiovascular events in hypertensive patients [12]. In our study, we found differences in cardiovascular and cerebrovascular mortality between groups with high and low levels of SII. A meta-analysis of 18 609 stroke participants showed that higher SII levels were significantly associated with poor outcome in stroke participants [43]. Furthermore, a cross-sectional study illustrated that SII was a valuable biomarker for forecasting cerebrovascular disease risk among individuals with asthma [44]. This may indicate that the SII has a greater predictive value in recognizing the risk of cerebrovascular disease. Further comprehensive research is required to investigate the impact of SII on diabetes.

### Strengths and Limitations

Our research demonstrates several strengths. Frist, NHANES collects various types of information, including health status, dietary intake, anthropometric measurements, biomarkers, and survey questionnaires. This allows us to conduct comprehensive analyses on the association between SII and all-cause mortality in DM and no-DM populations. Second, we used data from 8 cycles of NHANES, which offered a substantial nationally representative sample of individuals with diabetes for our analysis. However, our research had certain limitations. First, the data were obtained from single measurements and self-reports, which may introduce some biases. Self-reports reproduce recall bias. Participants may have errors in recalling past events or situations, leading to inaccurate information provided. Second, there are potentially confounding variables including treatment approach and dietary that were not adjusted in the study. Finally, the results need to be validated in other cohort study.

## Conclusion

This study uncovered a correlation between SII and diabetes. Furthermore, it was discovered that higher level of SII is connected to an elevated hazard of all-cause mortality among individuals with DM. This suggests that SII may serve as a potential biomarker for evaluating the risk of all-cause mortality in diabetic population.

## Data Availability

Some or all datasets generated during and/or analyzed during the current study are not publicly available but are available from the corresponding author on reasonable request.

## Abbreviations

BMI: body mass index
CVD: cardiovascular disease
DBP: diastolic blooding pressure
DM: diabetes mellitus
HDL-C: high-density lipoprotein cholesterol
HR: hazard ratio
LDL-C: low-density lipoprotein cholesterol
NHANES: National Health and Nutrition Examination Survey
NLR: neutrophil-to-lymphocyte ratio
PLR: platelet-to-lymphocyte ratio
SBP: systolic blooding pressure
SII: Systemic Immune-Inflammatory Index
TC: total cholesterol
TG: total triglyceride
